# The Anti-Obesogenic Effect of Lean Fish Species Is Influenced by the Fatty Acid Composition in Fish Fillets

**DOI:** 10.3390/nu12103038

**Published:** 2020-10-03

**Authors:** Even Fjære, Lene Secher Myrmel, Karianne Dybing, Ondrej Kuda, Benjamin Anderschou Holbech Jensen, Martin Rossmeisl, Livar Frøyland, Karsten Kristiansen, Lise Madsen

**Affiliations:** 1 Institute of Marine Research, NO-5817 Bergen, Norway; Even.Fjaere@hi.no (E.F.); LeneSecher.Myrmel@hi.no (L.S.M.); karianne@optimalfunksjon.no (K.D.); Livar.Froyland@hi.no (L.F.); 2 Department of Adipose Tissue Biology, Institute of Physiology of the Czech Academy of Sciences, 14220 Prague 4, Czech Republic; Ondrej.Kuda@fgu.cas.cz (O.K.); Martin.Rossmeisl@fgu.cas.cz (M.R.); 3 Novo Nordisk Foundation Center for Basic Metabolic Research, Faculty of Health and Medical Sciences, University of Copenhagen, DK-2200 Copenhagen, Denmark; benjamin.jensen@sund.ku.dk; 4 Department of Biology, University of Copenhagen, DK-2100 Copenhagen, Denmark; kk@bio.ku.dk

**Keywords:** nutrition, seafood, n-3 PUFA, EPA, DHA, phospholipids, endocannabinoids, marine protein source, obesity and mice

## Abstract

Fillets from marine fish species contain n-3 polyunsaturated fatty acids (PUFAs) in the form of phospholipids (PLs). To investigate the importance of PL-bound n-3 PUFAs in mediating the anti-obesogenic effect of lean seafood, we compared the anti-obesogenic properties of fillets from cod with fillets from pangasius, a fresh water fish with a very low content of PL-bound n-3 PUFAs. We prepared high-fat/high-protein diets using chicken, cod and pangasius as the protein sources, and fed male C57BL/6J mice these diets for 12 weeks. Mice fed the diet containing cod gained less adipose tissue mass and had smaller white adipocytes than mice fed the chicken-containing diet, whereas mice fed the pangasius-containing diet were in between mice fed the chicken-containing diet and mice fed the cod-containing diet. Of note, mice fed the pangasius-containing diet exhibited reduced glucose tolerance compared to mice fed the cod-containing diet. Although the sum of marine n-3 PUFAs comprised less than 2% of the total fatty acids in the cod-containing diet, this was sufficient to significantly increase the levels of eicosapentaenoic acid (EPA) and docosahexaenoic acids (DHA) in mouse tissues and enhance production of n-3 PUFA-derived lipid mediators as compared with mice fed pangasius or chicken.

## 1. Introduction

Nutritional strategies to curb the escalating public health problems precipitated by obesity and related co-morbidities are highly warranted. Human epidemiological studies suggest that diets with a high content of proteins from seafood may protect against the development of obesity [1,2,3]. This observation is supported by a number of rodent trials, where development of obesity and insulin resistance in seafood-fed animals is compared with animals fed meat from terrestrial sources [4]. The underlying mechanisms by which seafood intake is able to attenuate development of obesity and insulin resistance still remain to be fully elucidated. An anti-obesogenic potential of marine omega-3 polyunsaturated fatty acids (n-3 PUFAs) is suggested by several groups [5,6,7]. Meat from lean fish has a relatively low content of n-3 PUFAs, but appears to be as efficient as meat from fatty fish, rich in n-3 PUFAs, to attenuate obesity development [4].

Seafood protein is considered to be of high quality in terms of essential amino acid content. Compared to terrestrial protein sources, meat from seafood is characterized by high levels of the non-protein amino acid taurine [8] that has been demonstrated to attenuate both genetic [9] and diet-induced [10,11] obesity and insulin resistance. In addition to taurine, fish protein is rich in glycine. Both diets containing fish hydrolysates [12,13] and glycine supplementation [14] are reported to have anti-obesogenic effects and may increase energy expenditure by activation of brown adipocytes, possibly elicited by elevated levels of plasma bile acid [13]. In keeping with this notion, we previously demonstrated an inverse correlation between intake levels of taurine and glycine and adiposity in mice fed diets based on either chicken or different lean seafood protein sources [15].

Despite a low-fat content, lean marine fish species and seafood products based on such fish contain both eicosapentaenoic acid (EPA) and docosahexaenoic acids (DHA) [16]. Unlike fatty fish and cod liver oil, the majority of the marine n-3 PUFAs in shellfish and marine lean fish fillet is present in the form of phospholipids (PLs) [17,18,19]. PL-bound n-3 PUFAs have been considered as more bioavailable for tissue accretion than triacylglycerol (TAG)-bound n-3 PUFAs [20,21,22,23]. Intake of a Western diet with cod as the protein source led to increased levels of both EPA and DHA in mouse tissue phospholipids and attenuated obesity development [24]. However, supplementing a pork-based diet with PL-bound n-3 PUFAs extracted from herring roe did not protect against obesity in mice [19]. Still, in casein-based diets, the anti-obesogenic and anti-steatotic effects of PL-bound n-3 PUFAs [25], as well as the anti-atherosclerotic effects of PL-bound EPA in ApoE-/- mice [26], are reported to be superior to their TAG-bound counterparts. These findings [24,25] and other reports [27,28,29,30,31] collectively suggest that the mechanisms by which PL-bound n-3 PUFAs mediate their action are related to their ability to modulate production of PL-derived lipid mediators, such as endocannabinoids and eicosanoids, and to reduce hepatic lipid anabolism. 

To further investigate the importance of PL-bound n-3 PUFAs in mediating the anti-obesogenic effect of lean seafood, we took advantage of the fact that, unlike marine lean fish species, fillets of the lean, fresh water Asian shark catfish, pangasius (*Pangasius hypophthalmus*), have a higher content of n-6 than n-3 PUFAs in PLs, with a very low content of n-3 PUFAs [32]. We prepared high-fat/high-protein (HF/HP) diets using chicken, cod or pangasius as the protein sources, and-fed mice these diets for 12 weeks. Chicken meat was used as a non-fish-based n-6 PUFA-rich protein source. Although the sum of EPA and DHA comprised less than 2% of the total fatty acids in the cod-containing diet, this was sufficient to significantly increase the levels of marine n-3 fatty acids in mouse tissues and enhance production of n-3 PUFA-derived lipid mediators. Our results further demonstrate that cod was more efficient than pangasius to attenuate development of obesity and glucose intolerance. 

## 2. Materials and Methods

### 2.1. Ethical Statement

The mouse trial was approved by the Norwegian Animal Research Authority (Norwegian Food Safety Authority; FOTS id.nr 7882). Care, handling, and euthanasia were performed in accordance with national and international guidelines (Regulation on the use of animals in research, Ministry of Agriculture and Food, 1st July 2015; according to Directive 2010/63/EU of the European Parliament and of the Council of 22 September 2010).

### 2.2. Diets

The low-fat (LF) and high-fat/high-sucrose (HF/HS) reference diets were prepared using casein powder (C8654 SIGMA, Merck, Darmstadt, Germany) as earlier described [33]. Isoenergic high-fat/high-protein (HF/HP) experimental diets were prepared using fillets from frozen wild-caught Atlantic cod (Fiskemannen, Unil AS, Norway), pangasius (Godehav, Polar Seafood AS, Norway, or fresh skin free chicken breast fillets (Solvinge, Norsk kylling AS, Støren, Norway). The cod, pangasius and chicken fillets were heat treated (70 °C), freeze-dried and powdered. Fatty acid (Table 1) and amino acid (Table 2) compositions of the protein powder were measured as described [15], before the protein powder was added to the diets in amounts equal to 400 g crude protein/kg (Table 3), calculated from measurements of nitrogen content. Nitrogen was determined by the Dumas method using a Leco FP 628 nitrogen analyzer (Leco Corporation Svenska AB, Sweden), and we used Nx5.6 as the nitrogen to protein conversion factor [34]. The endogenous total fat content in the protein powders was determined gravimetrically after extraction with organic solvents before and after acidic hydrolysis [15], and the diets were adjusted with corn oil to ensure equal amounts of 250 g total fat/kg diet (Table 1). The diets were blended using a Crypto Peerless EF20 blender and analyzed for gross energy by bomb calorimetry (Parr Instrument, Moline, IL, USA).

### 2.3. Mouse Study

We obtained 50 male C57BL/6J BomTac mice, at 8 weeks of age, from Taconic (Ejby, Denmark). The mice were single housed in individually ventilated cages (IVC) in a thermoneutral environment (29 ± 1 °C) with 50% relative humidity and on a 12:12 h light–dark cycle. Prior to the experiment, the mice were acclimated for one week and provided the low-fat reference diet and fresh water ad libitum. The mice were subjected to a non-invasive scanning using a time-domain nuclear magnetic resonance system (Bruker Minispec LF50 Body Composition Analyzer mq 7.5 (Bruker Optik GmbH, Ettlingen, Germany)) and distributed into 5 experimental groups (*n* = 10) in a manner to equalize group means of total, lean, and fat mass. During the experiment, mice had free access to water and were fed their respective diets. Fresh water was provided twice per week and fresh feed three times per week. Body weight was recorded once per week and feed intake was recorded three times per week. In week 9, we replaced the wooden bedding in the cages with a paper cover and collected feces to measure apparent digestibility of protein and fat, see Section 2.4. At the end of week 9, whole-body composition, fat mass, lean mass, and free water were remeasured by NMR, as described above. We performed an insulin tolerance test (Section 2.5) and an oral glucose tolerance test (Section 2.6) in week 10 and 11, respectively. After 12 weeks, the mice were euthanized by cardiac puncture under isoflurane anesthesia (Isoba vet, Schering-Plough, Ballerup, Denmark). Blood was collected from the heart into tubes containing EDTA, and red blood cells were isolated by centrifugation (1500 G, 15 min, 4 °C) and stored at −80 °C until further analyses. Liver and adipose tissues were dissected out, weighed, freeze-clamped in liquid nitrogen and stored at −80 °C until further analyses.

### 2.4. Apparent Digestibility of Nitrogen and Fat

During the 9th week of the experiment, the mice were placed in cages with a paper cover. All feces were collected from the cages, weighed and frozen at −20 °C until analyzes. Total fat in feces was determined gravimetrically after extraction with organic solvents before and after acidic hydrolysis and nitrogen using Dumas method, as described above. Apparent digestibility of nitrogen and fat was calculated using the formula: 100 × [intake (mg) − feces output (mg)/(intake (mg)].

### 2.5. Insulin Tolerance Test (ITT)

After 10 weeks, each mouse was injected intraperitoneally with 1.0 U insulin/kg lean mass in the fed state. Glucose was measured prior to insulin injection at baseline and after 15, 30, 45 and 60 min in blood collected from the tail vein using a glucometer (Ascensia Contour, Bayer, Norway). The area over the curve (AOC) was calculated.

### 2.6. Glucose Tolerance Test (GTT)

After 11 weeks, each mouse was fasted for five hours. An oral dose of 3 mg glucose/g lean mass was given by gavage. Blood glucose was measured prior gavage at baseline and after 15, 30, 60 and 120 min, as described above. The incremental area under the curve (iAUC) for the OGTT was calculated using the formula: iAUC = AUC − (basal glucose*120 min). At baseline, and 15 min after gavage, additional 20 µL blood were collected to prepare 5 µL plasma for insulin measurements (Ultra sensitive Mouse Insulin ELISA Kit, Crystal Chem (Europe), Zaandam, The Netherlands.

### 2.7. Histology and Immunohistochemistry

Parts of iBAT and iWAT were fixed in 4% formaldehyde in 0.1 M phosphate buffer (PB) overnight. The tissue was then rinsed once in PB, gradually dehydrated in increasing concentration of alcohol, cleared in xylene and embedded in paraffin blocks as earlier described [35]. Sectioning was performed at the Molecular Imaging Center (MIC) at the University of Bergen. The 5 μm thick sections were stained with Hematoxylin and Eosin for morphology investigations. Adipocyte cell size was determined and immunohistological detection of UCP1-positive cells was performed by an avidin-biotin peroxidase method, as described earlier [35].

### 2.8. Real Time qPCR

RNA was extracted by homogenization of liver tissue together with Trizol reagent (Invitrogen). The RNA quantity was evaluated using the NanoDrop ND-1000 UV–Vis Spectrophotometer (Saveen & Werner, Limhamn, Sweden), and RNA quality was tested on a random selection of samples using a BioAnalyzer—RNA 6000 Nano (Agilent Technologies, Santa Clara, CA, USA). Reverse transcription and real-time qPCR analysis were performed as described [36]. Primer sequences are available on request.

### 2.9. Lipid Analyses

Lipids were extracted from the diets, mouse livers, red blood cells and epididymal white adipose tissues by adding 20 × the amount of sample (*v/w*) of chloroform:methanol (2:1). To separate polar and neutral lipids in the diets and liver, solvents were evaporated and the residue was dissolved in 2% methanol in chloroform and separated using solid-phase extraction (SPE). After filtration of the extract, the SPE cartridge (Biotage Isolute SI 500 mg/10mL) was conditioned by 5 mL of hexane. The sample was then loaded and eluted with 10 mL 2% methanol in chloroform, and the neutral fraction was collected. Then 15 mL methanol were added and the fraction was collected as the polar lipids. The methyl ester of C19:0 (non-adecanoic acid) was added to each fraction/sample as the internal standard before saponifying the lipid samples with NaOH and methylating the fatty acids using 12% BF_3_ in methanol. Fatty acids in each fraction were determined by a gas chromatography (GC) (GLC TRACE GC 2000, Thermo Fisher Scientific, Waltham, MA, USA) column (CP-sil-88, 50 m WCOT) coupled with a flame ionization detector, identified by retention time using standard mixtures of methyl esters (Nu-Chek Prep, Elysian, MN, USA) and quantified towards the internal standard under conditions as previously described [19] based on Lie et al. [17]. Limit of quantification was 0.01 mg fatty acids/g sample. As red blood cells and white adipose tissue comprise mainly polar and neutral lipids, respectively, lipids from these tissues were not separated prior methylation and quantification in GC.

### 2.10. Plasma and Liver Analyses

EDTA plasma and liver samples for analysis of oxylipins and endocannabinoids were prepared in methanol containing 1 µM butylated hydroxytoluene, (Sigma #47168) and protease inhibitors; 1 µM soluble epoxide hydrolase inhibitor, (Cayman # 10007927), 1 µM monoacyl glycerol lipase inhibitor (Cayman # 10007457), 1 µM omega-hydrolase inhibitor (Cayman # 10018) and 1 µM CYP450 inhibitor (Cayman # 75770). Lipids were extracted using Strata-X SPE columns and analyzed with a UPLC system (UltiMate 3000 Binary RSLC System, Thermo) coupled to a Qtrap 5500 (AB-Sciex, Foster City, CA, USA) mass spectrometer using multiple reaction monitoring as earlier described [24,37].

### 2.11. Statistics

Mice fed the low-fat diet were used as the reference of normal weight and health development, whereas mice fed HF/HS were used as a reference for development of obesity and reduced glucose tolerance and insulin sensitivity. Hence, only mice fed a HF/HP diet based on different protein sources were included in the statistical analyses. All data are presented as the mean ± SEM. Homogeneity of variances of the data was examined by Brown–Forsythe test and the data were compared between groups using one-way ANOVA followed by Fisher’s LSD multiple comparison post hoc test. Data demonstrating differences in variance by the Brown–Forsythe test data were analyzed by the non-parametric Kruskal–Wallis test. Growth curve and glucose levels during insulin and glucose tolerance testing were analyzed by repeated-measure one-way ANOVA and Fisher’s LSD multiple comparison post hoc test. Group means were considered statistically different at *p* < 0.05. Statistical analyses were performed using Graph Pad Prism version 8.3.0 (GraphPad software, La Jolla, CA, USA).

## 3. Results

### 3.1. A High-Fat/High-Protein Diet Containing Cod is More Efficient than An Isoenergic Diet Containing Pangasius in Attenuating Development of Obesity and Glucose Intolerance

**Figure 1:**
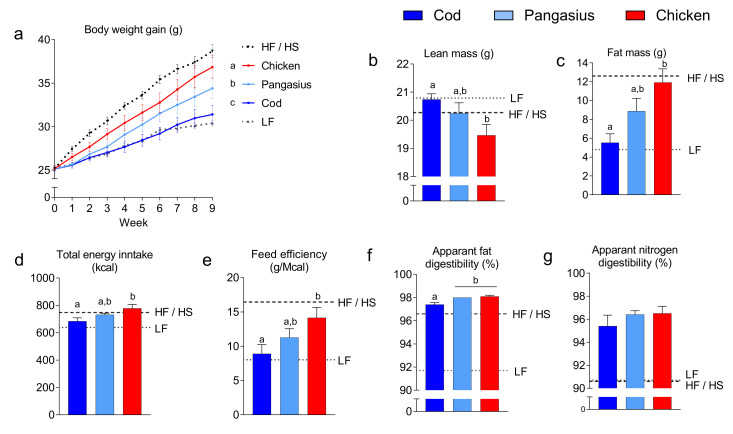
(**a**) Body weight development, (**b**) fat mass and (**c**) lean mass after 9 weeks of ad libitum feeding high-fat/high-protein (HF/HP) diets based on the different protein sources, in addition to one group fed a low-fat (LF) reference diet and one group fed an obesogenic high-fat/high-sucrose (HF/HS) diet. (**d**) Total energy intake during 9 weeks of feeding. (**e**) Feed efficiency (g/Mcal) from 9 weeks of feeding, calculated as g increased body weight per Mcal eaten. (**f**,**g**) Apparent fat and nitrogen digestibility measured during the experiment. Data are presented as the mean ± SEM (*n* = 10) and different letters denote significant differences (*p* < 0.05) by one-way ANOVA using uncorrected Fisher’s LSD multiple comparison. Body weight development was analyzed by repeated-measure one-way ANOVA and Fisher’s LSD multiple comparison post hoc test.

We first compared the obesogenic potential of diets based on cod, pangasius or chicken, where chicken represents an obesogenic protein source [33]. Hence, we fed C57BL/6J mice isocaloric HF/HP diets containing cod, pangasius or chicken for 12 weeks. As references, two groups of mice were fed either a low-fat (LF) diet or a standard high-fat/high-sucrose (HF/HS) diet. In accordance with earlier observations [33], mice fed cod gained less weight than mice fed chicken (Figure 1a). Weight gain in mice fed the HF/HP chicken diet was comparable to the weight gain observed in the HF/HS casein fed reference mice. By contrast, we found weight gain in mice fed the HF/HP cod diet comparable to the weight gain observed in the LF diet fed reference mice. Weight gain in mice fed the HF/HP pangasius diet was increased compared to mice fed cod, but reduced compared to mice fed chicken. 

NMR scanning (Figure 1b,c) confirmed that the differences in body mass were mainly due to differences in body fat. The differences in fat mass between mice fed HF/HP diets containing cod, pangasius or chicken reflected the masses of the different adipose tissue depots (Figure S1). Although total energy intake was higher in mice fed the HF/HP chicken diet than in mice fed the HF/HP cod diet, we still observed notable differences in feed efficiency (Figure 1d,e), suggesting that differences in obesity were not solely related to energy intake.

To evaluate whether the observed differences in feed efficiency were a consequence of altered digestibility, we measured the apparent digestibility of nitrogen and fat. The apparent digestibility of fat was slightly lower in mice fed the HF/HP cod diet than in mice fed the HF/HP pangasius or the HF/HP chicken diets (97.4 vs. 98 and 98.1%), whereas no significant differences in apparent nitrogen digestibility were observed (Figure 1f,g). Thus, lower feed intake and reduced fat digestibility may contribute to reduce fat mass in mice fed cod.

**Figure 2:**
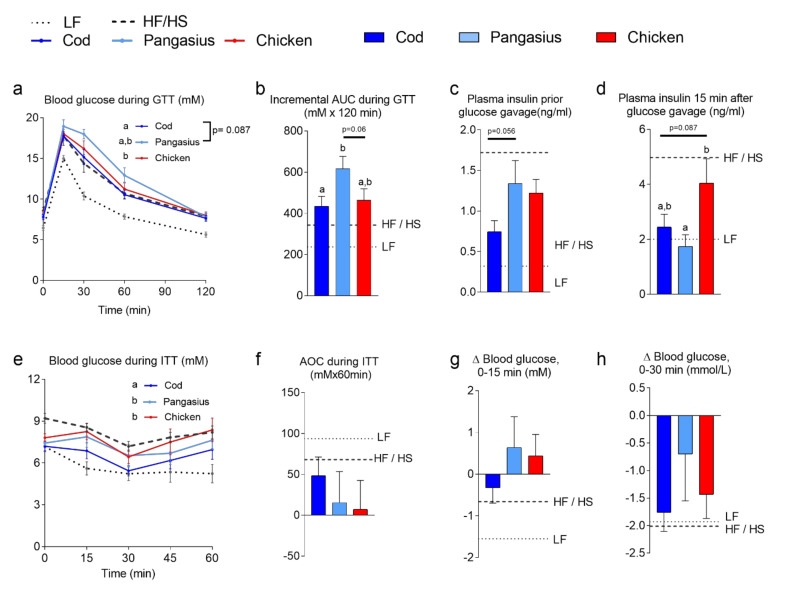
(**a**) Blood glucose levels during an oral glucose tolerance test (OGTT) performed in week 10 and (**b**) calculated incremental area under the curve (AUC) for the OGTT. (**c**) Plasma insulin levels after 5 h fasting prior to glucose injection. (**d**) Plasma insulin levels 15 min after glucose injection. (**e**) Blood glucose levels during the insulin tolerance test (ITT) in week 11 and (**f**) calculated decremental area over the curve (AOC) for the ITT. (**g**,**h**) Change in blood glucose from the fed state to 15 min (g) and 30 min (h) after insulin injection. Data are presented as the mean ± SEM (*n* = 10) and different letters denote significant differences (*p* < 0.05) by one-way ANOVA using uncorrected Fisher’s LSD multiple comparison. Blood glucose data during OGTT and ITT were analyzed by repeated-measure one-way ANOVA and Fisher’s LSD multiple comparison post hoc test.

To investigate whether the differences in obesity development led to differences in insulin sensitivity and glucose tolerance, we performed glucose and insulin tolerance tests at week 10 and 11, respectively. The glucose tolerance test and calculated incremental AUC revealed that mice fed HF/HP pangasius were less glucose tolerant than mice fed HF/HP cod (Figure 2a,b). Five hours fasting plasma insulin levels tended to be lower (*p* = 0.056) in HF/HP cod than HF/HP pangasius-fed mice (Figure 2c). HF/HP chicken-fed mice further exhibited elevated insulin secretion 15 min post glucose gavage, compared to their HF/HP pangasius-fed counterparts (Figure 2d). We next performed an insulin tolerance test on animals in the fed state, and measured blood glucose 15, 30, 45 and 60 min after insulin injection. Repeated measurement analyses revealed significantly better insulin response on blood glucose levels in cod, than pangasius- and chicken-fed mice (Figure 2e), although no significant differences in decremental area over the curve the first 15 and 30 min (first phase insulin secretion) were observed (Figure 2f–h). Together, these data indicate that glucoregulatory disturbances did not necessarily mirror obesity development.

### 3.2. Mice fed a HF/HP Diet with Cod had Smaller Adipocytes than Mice fed a HF/HP Diet with Pangasius or Chicken

**Figure 3:**
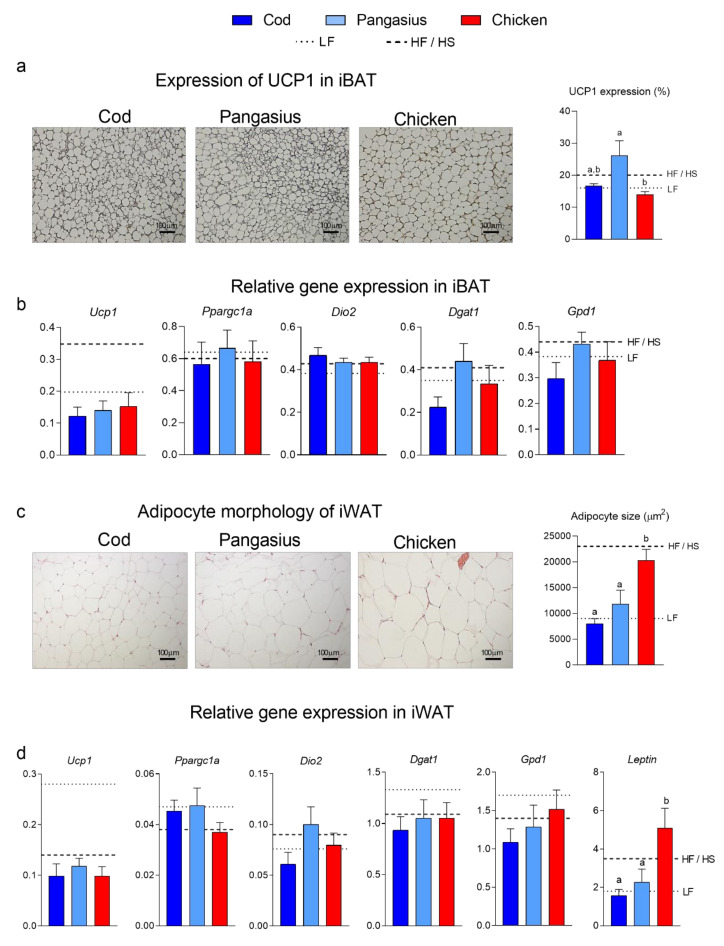
(**a**) Micrographs of immunohistochemically stained iBAT and quantified UCP1 expression (scalebar = 100 μm). (**b**) Relative expressions of uncoupling protein 1 (*Ucp1*), peroxisome proliferator activated receptor gamma co-activator 1 alpha (*Ppargc1a*), iodothyronine deiodinase 2 (*Dio2*), diglyceride acyltransferase 1 *(Dgat1*), glyceraldehyde-3-phosphate dehydrogenase 1 (*Gpd1*) measured by RT-qPCR in iBAT. (**c**) Adipocyte micrographs and mean adipocyte size of H&E-stained iWAT (scalebar = 100 μm). (**d**) Expressions of *Ucp1*, *Ppargc1a*, *Dio2*, *Dgat1* and *Leptin* were measured by RT-qPCR in iWAT. Data are presented as the mean ± SEM (*n* = 10) and different letters denote significant differences (*p* < 0.05) by one-way ANOVA using uncorrected Fisher’s LSD multiple comparison.

The differences in amino acid composition in freeze-dried protein from cod, pangasius and chicken, and hence the diets, are subtle (Table 1). Protein from pangasius had 4.5% and 7.4% higher content of branched-chain amino acids than proteins from chicken and cod, respectively (Pangasius: 166.5, Cod: 159.3, and chicken: 155.0 mg/g). Further, the freeze-dried pangasius muscle had higher content of hydroxyproline than chicken and cod (3.6, 1.7 and 1.5 mg/g, respectively). Moreover, the levels of taurine in freeze-dried protein from cod, pangasius and chicken were 4.8, 2.6 and 0.6 mg/g (Table 1). The recent notion that taurine may lead to increased energy expenditure and activation of UCP1 [10] prompted us to investigate the appearance of iBAT in mice fed cod, pangasius and chicken. The brown phenotype was, however, not apparent in any mice and large fat droplets were observed in all groups (Figure 3a). Accordingly, UCP1 expression measured by immunohistochemistry was low. UCP1 expression was higher in iBAT from pangasius- than chicken-fed mice (Figure 3a). However, expression of brown adipocyte marker genes and genes involved in triacylglycerol synthesis was similar in mice fed the three diets (Figure 3b).

In accordance with iWAT masses, adipocytes in chicken-fed mice were larger than adipocytes in mice fed cod and pangasius. The sizes of adipocytes in the pangasius-fed mice was not significantly different from cod-fed mice (Figure 3c). Expression of genes involved in thermogenesis and triacylglycerol synthesis in iWAT was not significantly different in mice fed the three diets, but expression of leptin mirrored adipocyte size and adipose tissue mass and was higher in mice fed the chicken-based diet that in mice fed the cod- or the pangasius-based diets (Figure 3d).

### 3.3. Feeding Mice a HF/HP Diet with Cod Increases Incorporation of Marine n-3 Fatty Acids in Mice Tissues

**Figure 4:**
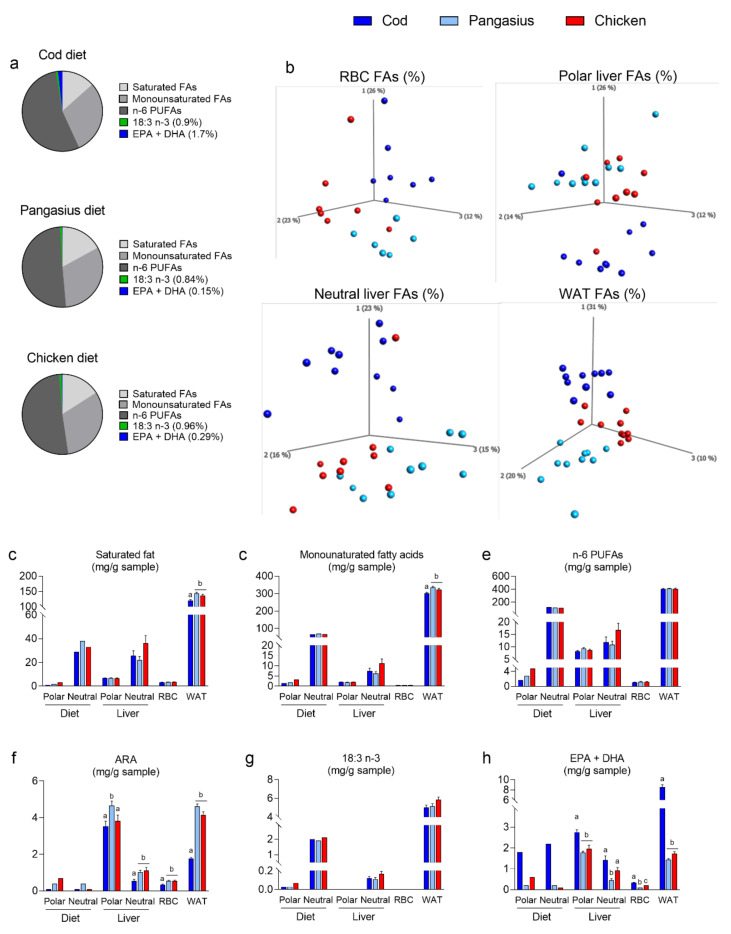
(**a**) Fatty acid composition of the HF/HP diets with cod, pangasius or chicken as the protein source. (**b**–**h**) Lipids from diets and livers were extracted and separated into polar and neutral lipid fractions. Fatty acid composition was measured and quantified in the fractions from diets, livers (*n* = 10), in extracted lipids from red blood cells comprising mainly polar lipids (RBCs) (*n* = 7) and in white adipose tissue (eWAT) comprising mainly neutral lipids (WAT) (*n* = 10). (**b**) PCA plot of fatty acid composition (%) in RBCs, polar liver lipids, neutral liver lipids and eWAT. (**c**) Sum saturated fatty acids, (**d**) sum monounsaturated fatty acids, (**e**) sum n-6 fatty acids, (**f**) sum arachidonic acid (ARA, 20:4 n-6), (**g**) sum alpha-linolenic acid 18:3 (n-3) and (**h**) sum eicosapentaenoic acid (EPA, 20:5 n-3) + docosahexaenoic acid (DHA, 22:6 n-3). Data from livers, RBCs, and WAT are presented as the mean mg fatty acids/g sample ± SEM and analyzed using one-way ANOVA followed by Fisher’s LSD post hoc test. Different letters denote statistical significance (*p* < 0.05) between the groups.

Freeze-dried fillets from cod, pangasius and chicken contain 17.4, 62.7 and 78 mg lipids/g fillet, respectively (Table 1). As expected, freeze-dried cod contained more marine n-3 PUFAs than freeze-dried pangasius and chicken (Table 1). The HF/HP diets contained 250g fat/kg and fat from the protein sources comprised 3.3, 12.6 and 16.0 w% of the total fat content in HF/HP diets with cod, pangasius and chicken, respectively (Table 3). Hence, as illustrated in Figure 4a, the marine n-3 fatty acids comprised a small fraction of the total fatty acid composition of the diets, 1.7, 0.15 and 0.29%, of the cod, pangasius and chicken-containing diets, respectively. As the main fat source in the diets was corn oil, all diets contained a large proportion of n-6 PUFAs in addition to monounsaturated and saturated fatty acids (Figure 4a). We next aimed to investigate how 12 weeks of feeding influenced the fatty acid composition of liver, white adipose tissue (WAT) and red blood cells (RBCs) in the mice.

As demonstrated by PCA plots, the fatty acid composition in organs collected from mice fed chicken and pangasius were more similar than the fatty acid composition in organs collected from cod-fed mice (Figure 4b). WAT and RBCs contain mainly neutral and polar lipids, respectively. However, as the liver contains both a polar and neutral fraction, liver lipids were fractionated before fatty acid composition measurements. As confirmed here, monounsaturated and saturated fatty acids were primarily found in neutral lipids in mouse tissue (Figure 4b–d). In line with lower adipose tissue mass, the levels of saturated and monounsaturated fatty acids were lower in WAT from HF/HP cod-fed mice than in pangasius- and chicken-fed mice (Figure 4c,d). All diets contained a high proportion of n-6 PUFAs in the neutral fraction, and the total n-6 PUFAs levels in mice liver fractions, WAT, and RBCs were not influenced by the diet. However, arachidonic acid (ARA) levels in the polar lipid fraction were higher in HF/HP pangasius-fed mice than in cod-fed mice. The levels of ARA in organs collected from pangasius-fed mice were in fact as high, or even higher than levels measured in chicken-fed mice (Figure 4f). The levels of alpha-linolenic acid (ALA), the main n-3 PUFA found in vegetable sources, were similar in organs from cod-, pangasius- and chicken-fed mice, whereas levels of the marine n-3 PUFAs (EPA + DHA) were significantly higher in organs collected from cod than pangasius-fed mice (Figure 4g,h).

**Figure 5:**
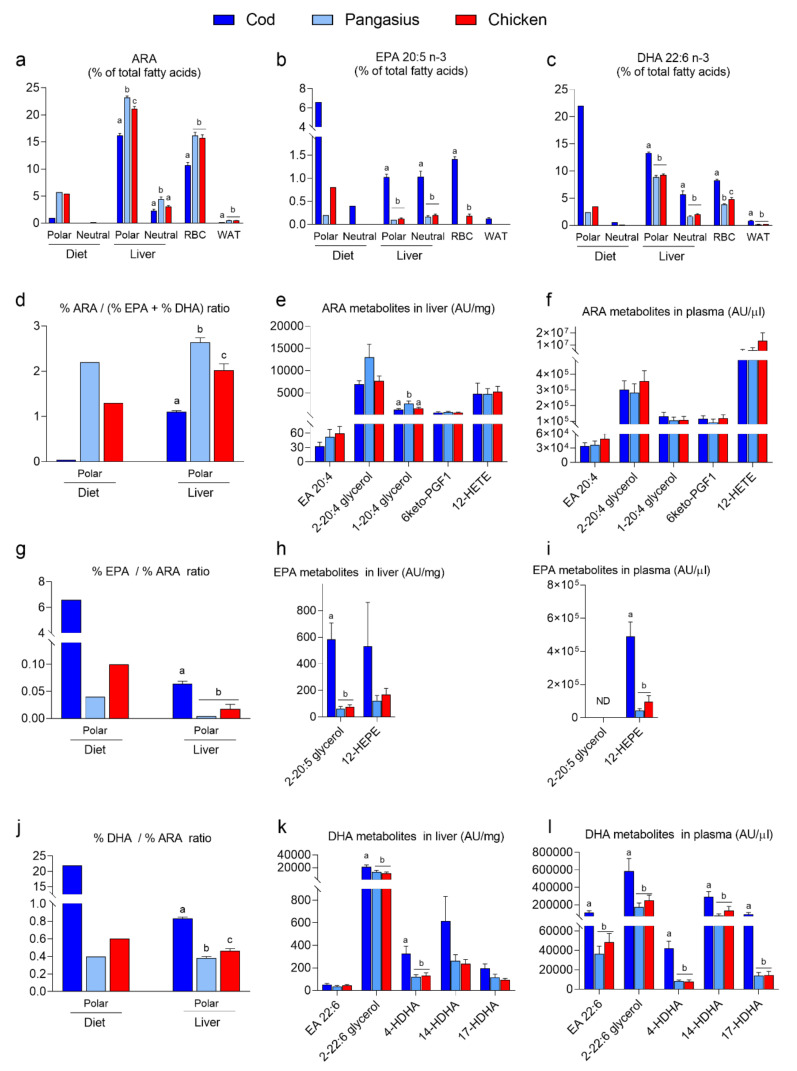
Relative proportion of (**a**) arachidonic acid (ARA), (**b**) eicosapentaenoic acid (EPA) and (**c**) docosahexaenoic acid (DHA) in neutral and polar fractions of HF/HP diets, neutral and polar fractions of mice livers (*n* = 10), red blood cells (RBCs) and white adipose tissue (WAT). (**d**) Relative ARA:(EPA+DHA) ratio in polar lipid fractions of diets and livers. (**e**,**f**) ARA-derived lipid mediators; anandamide (EA 20:4), 2-arachidonoyl glycerol (2-20:4 glycerol), 1-arachidonoyl glycerol (1-20:4 glycerol), 6-keto PGF_1_, and 12-HETE in livers (**e**) and plasma (**f**). (**g**) Relative EPA:ARA ratio in polar lipid fractions of diets and livers. (**h**,**i**) EPA-derived lipid mediators; 2-eicosapentanoyl-glycerol (2-20:5 glycerol) and 12-HETE in livers (**h**) and plasma (**i**). (**j**) Relative DHA:ARA ratio in polar lipid fractions of diets and livers. (**k**,**l**) DHA-derived lipid mediators; docosahexaenoyl ethanolamide (EA 22:6), docosahexanoyl-glycerol (2-22:6 glycerol), 4-HDHA, 14-HDHA, 17-HDHA, in livers (**k**) and plasma (**l**). Data from livers, RBCs, WAT, and plasma are presented as the mean ± SEM and analyzed using one-way ANOVA followed by Fisher’s LSD post hoc test. Different letters denote statistical significance (*p* < 0.05) between the groups.

ARA, EPA and DHA are essential constituents of cell membranes in all muscle PLs, and the dietary levels of these fatty acids mirrored the levels measured in the fillet of cod, pangasius and chicken meat (Figure 4f–h). The fat content of the diets was approximately 90% neutral fat. Hence, the relative proportions (%) of ARA, EPA and DHA in the diets are by far higher in the polar lipid fraction than the neutral lipid fraction. (Figure 5a–c). The relative proportions of both EPA and DHA were higher, whereas ARA levels were lower, in tissues from mice fed HF/HP cod than in pangasius- or chicken-fed mice (Figure 5a–c). When EPA and DHA are incorporated into phospholipids, they compete with ARA as substrates for enzymes producing eicosanoids. The lower ARA: (EPA+DHA) ratio in the polar lipid fraction of the cod diet led to lower ARA: (EPA+DHA) ratio in hepatic polar lipids of cod-fed mice (Figure 5d). Still, most of the ARA metabolites measured in liver and plasma were comparable between all HF/HP-fed mice, regardless of the dietary protein source (Figure 5e,f). The hepatic levels of the ARA-derived endocannabinoids, 1-, and 2-arachidonoylglycerol (2-AG), appeared to mirror the ARA: (EPA+DHA) ratio, but only levels of 1-arachidonoylglycerol were significantly higher in pangasius-fed mice (Figure 5e). However, higher EPA:ARA and DHA:ARA ratios in the polar fraction of the cod diet and polar lipids in liver from cod-fed mice led to higher levels of EPA- and DHA-derived metabolites in both liver and plasma (Figure 5g–l). Notably, the levels of EPA-derived eicosapentanoyl-glycerol (2-20:5 glycerol) and the DHA-derived docosahexaenoyl ethanolamide (EA 22:6) and docosahexanoyl-glycerol (2-22:6 glycerol) were significantly higher in liver and/or plasma in mice fed the cod diet, compared with mice fed pangasius or chicken. Similarly, the levels of 5- and 12-LOX metabolites from DHA, 4-HDHA and 14-DHA, as well as 17-HDHA were increased in both liver and plasma from cod-fed mice (Figure 5k,l).

## 4. Discussion

In line with human epidemiological studies [1,2,3] and earlier observations in mice [15,24,33,38,39,40], we here demonstrate that different protein sources exhibit profound differences in their ability to modulate obesity development. This study supports the notion that dietary proteins from marine sources are less obesogenic than protein from terrestrial sources [24,38,39]. Thus, mice fed the chicken-based diet gained more weight than mice fed the cod-based diet, whereas weight gain in mice fed the pangasius-based diet fell in between.

Mice fed diets with cod as the protein source had lower adipose tissue mass than mice fed chicken. The reduced white adipose tissue mass in cod-fed animals was accompanied with a lower adipocyte size and reduced expression of *Leptin*. Fat mass acquisition in mice fed pangasius was intermediate between the cod- and chicken-fed mice. Further, compared with cod-fed mice, mice fed the pangasius-based diet had reduced glucose tolerance.

The fillets of most fresh water fish, including pangasius, have a higher content of n-6 than n-3 PUFAs compared to marine lean fish species, and the fatty acid profile in pangasius fillets is more similar to the fatty acid profile in chicken than in cod. In addition, the level of marine n-3 PUFAs in the phospholipid fraction is very low in pangasius. Cod, pangasius and chicken are all lean protein sources and fat from the protein sources represented less than 20% of the total fat in the chicken- and pangasius-based diets, and less than 4% in the cod-containing diets. Hence, the fatty acid profiles in all experimental diets were dominated by corn oil. Still, fatty acid composition in red blood cells, liver and white adipose tissue in mice fed cod were different from organs collected from chicken and pangasius-fed mice. In particular, mice fed the cod-containing diet had a lower level of ARA and higher levels of the marine n-3 PUFAs, EPA and DHA in the analyzed organs. In cod-fed animals, the highest levels of EPA and DHA were found in WAT. However, when examining the relative proportions of the individual fatty acids, higher proportions of EPA and DHA in polar lipids were prominent in all analyzed organs from cod-fed mice.

The cod-based diet in this study provided 4.5 mg EPA and DHA per g diet, thus far below studies demonstrating anti-obesogenic effects, where doses are ranging from 60 up to 350 mg/g diet [29,41,42,43,44,45,46,47]. However, the relative proportion of EPA and DHA in the polar fraction of the cod-based diet comprised > 6 and 22%, respectively, of the total fatty acids. Given that a large fraction of the n-3 PUFAs in cod fillets is present in phospholipids, our results are in line with the reported higher bioavailability of PL-bound relative to TAG-bound EPA and DHA [20,21,22,23,25]. However, it has also been reported that dietary PL-bound n-3 PUFAs and TAG-bound n-3 PUFAs have similar bioavailability [19] and a critical review published in 2014 concluded that there was no evidence for greater bioavailability of n-3 PUFAs from PLs compared with TAGs [48]. Still, results from this study are in line with other studies demonstrating that an increased n-3:n-6 ratio in fish fillets and in PLs from livers and RBCs collected from mice consuming fish-containing diets, associates with reduced obesity [24,49,50,51]. Together, these data indicate that small differences in phospholipid composition in the fish fillet, which often differentiate fresh water fish from marine fish, may have an impact on obesity development. However, differences in obesity development related to the different fatty acid profiles in the diets did not directly translate into differences in insulin sensitivity and glucose tolerance.

The reported high biological activity of PL-bound PUFAs is suggested to be mediated via the endocannabinoid signaling system [25,27]. Competition between n-3 PUFAs and ARA for incorporation into PLs may affect substrate availability for syntheses of the two major endogenous endocannabinoids 2-arachidonoyl glycerol (2-AG) and anandamide (AEA). However, the higher relative ARA/(EPA+DHA) ratio in the polar lipid fraction in livers from pangasius- and chicken-fed mice than cod-fed mice did not result in higher levels of neither 2-AG nor AEA. Still, in line with earlier observations [25,29,52], higher relative EPA/ARA and DHA/ARA ratios in the polar liver lipid led to increased substrate availability for formation of the EPA- and DHA-derived endocannabinoid-like molecules. We observed higher levels of both eicosapentanoyl-glycerol (2-20:5 glycerol), docosahexaenoyl ethanolamide (EA 22:6) and docosahexanoyl-glycerol (2-22:6 glycerol) in liver and/or plasma in mice fed the cod diet, compared with mice fed pangasius or chicken. Given the importance of the cannabinoid receptor CB1 in diet-induced obesity [53,54,55] and feed intake [56,57], modulation of the endocannabinoid tonus may represent a mechanism whereby cod may attenuate obesity development. We did not observe increased expression of UCP1 and browning of white adipose tissue as observed when CB1 is ablated or blocked [58,59], but the increased circulating levels of EPA and DHA-derived endocannabinoids in cod-fed mice were accompanied by reduced cumulative energy intake. Hence, cod may attenuate obesity development via reduced energy intake mediated by a reduced endocannabinoid tone. Of note, however, feed efficiency calculated as weight gain per Mcal eaten was significantly lower in cod-fed mice than chicken-fed mice.

Compared with terrestrial protein sources, marine protein sources are characterized by high levels of taurine [8]. The level of taurine in the freshwater fish pangasius was higher than in chicken, but lower than in cod. Hence, in line with the study by Tastesen et al. [15], the obesogenic effect mirrored levels of dietary taurine. It is well established that taurine attenuates obesity development by enhancing energy expenditure. It is uncertain, however, whether WAT or BAT is involved. Kim et al. reported that inclusion of 2% taurine in the drinking water increased expression of thermoregulatory genes in BAT, but not WAT [10], whereas Guo et al. demonstrated that taurine supplementation induced browning of iWAT accompanied with significantly elevated expression of *Ucp1*, *Ppargc1a* and other thermogenic genes [60]. Here, we did not observe an increased expression of neither *Ucp1* nor *Ppargc1a*, in either BAT or WAT from cod-fed animals, but we cannot exclude the possibility that taurine contributes to the anti-obesogenic properties in the cod-fed mice. Similarly, the higher content of taurine in pangasius compared to chicken may also contribute to the slightly lower weight gain in mice fed the pangasius-containing diet compared to mice fed the chicken-based diet.

In conclusion, compared with mice fed a HF/HP diet based on chicken meat, mice fed cod exhibited reduced obesity development. Mice fed pangasius generally presented an intermediate phenotype, but had reduced glucose tolerance compared with cod-fed animals. The fatty acid composition in pangasius fillets resembles chicken fillets, whereas cod fillets have a higher amount of EPA and DHA. Despite a low-fat content in fillets from cod, pangasius and chicken, mice fed the fillets had altered fatty acid composition in RBCs, liver and WAT. Replacement of ARA with EPA and DHA in the hepatic polar fraction of cod-fed mice translated into an increased production of n-3 PUFA-derived lipid mediators as compared with mice fed pangasius and chicken, and differences in the production of EPA- and DHA-derived lipid mediators may contribute to the differences in obesity development between the diets.

## Figures and Tables

**Table 1:** Fatty acid composition in the neutral and polar lipid fractions from freeze-dried protein sources used in the experimental diets.

FA (mg/g)	Cod	Pangasius	Chicken	Cod	Pangasius	Chicken
	Neutral Lipid Fraction ^5^			Polar Lipid Fraction ^6^		
Sum total FAs ^1^	10	50	60	8	12	18
Sum SFAs ^2^	2.3	20.3	18.1	2.0	3.1	5.4
Sum MUFAs ^3^	1.9	19.9	27.2	1.3	2.9	4.3
18:1 n-9	0.7	18.2	22.6	0.6	2.5	3.4
Sum n-6 PUFAs ^4^	0.4	7.6	12.5	0.3	3.9	5.1
18:2 n-6	0.1	5.3	11.8	0.1	1.1	2.8
20:4 n-6	0.2	0.7	0.3	0.1	1.1	1.5
Sum n-3 PUFAs^4^	5.46	0.83	1.48	4.31	0.68	1.56
18:3 n-3	0.26	0.28	1.04	0.01	0.03	0.06
20:5 n-3	1.76	0.06	0.05	0.87	0.04	0.17
22:6 n-3	3.40	0.21	0.09	3.24	0.44	0.82

Total fat levels in freeze-dried protein from cod, pangasius and chicken were 17.4, 62.7 and 78.0 mg/g, respectively. ^1^ Fatty acids (FAs), ^2^ saturated fatty acids (SFAs), ^3^ monounsaturated fatty acids (MUFAs), and ^4^ polyunsaturated fatty acids (PUFAs). ^5^ The neutral lipid fraction comprises triacylglycerols (TAGs), diacylglycerols, cholesteryl esters and cholesterol. ^6^ The polar lipid fraction is composed of phospholipids (>85%), sphingolipids and glycerolipids.

**Table 2:** Amino acid composition in freeze-dried protein sources used in the experimental diets.

Amino acids (mg/g)	Cod	Pangasius	Chicken
*Indispensable*			
Leucine	72.8	76.1	70.4
Isoleucine	40.3	43.6	40.7
Valine	46.3	46.8	43.9
Lysine	93.1	94.6	85.3
Methionine	28.3	27.7	24.7
Phenylalanine	34.6	38.1	34.9
Threonine	39.6	43.1	39.6
Tryptophan	9.5	9.9	10.3
Histidine	16.9	19.5	23.7
*Dispensable*			
Alanine	54.0	52.7	50.9
Arginine	53.7	56.9	53.8
Aspartate	101.8	101.0	89.0
Cysteine	1.0	1.0	1.0
Glutamate	146.9	148.3	135.2
Glycine	38.4	42.3	36.4
Proline	30.0	32.8	30.8
Serine	39.4	38.5	35.1
Tyrosine	30.0	31.5	28.9
Sum BCAAs ^1^	159.3	166.5	155.0
Hydroxyproline	1.5	3.6	1.7
Taurine	4.8	2.6	0.6

^1^ Branched-chain amino acids (BCAAs).

**Table 3:** Dietary composition of LF ^1^, HF/HS ^2^ and HF/HP ^3^ diets.

Component (g/kg)	LF	HF/HS	HF/HP Cod	HF/HP Pangasius	HF/HP Chicken
Casein ^#^	207	207			
Cod ^#^			482		
Pangasius ^#^				502	
Chicken ^#^					501
Protein from protein source *	200	200	400	400	400
Fat from protein source **	1	1	8.4	31.5	40.0
Corn oil	69	249	242	219	210
Sucrose	92	439	140	140	140
Dextrin	531	5	36.0	38.7	48.2
L-cystine	3	3	3	3	3
Cellulose	50	50	50	50	50
t-butylhydroquinone	0.01	0.01	0.01	0.01	0.01
Mineral mix ^†^	35	35	35	35	35
Vitamin mix ^‡^	10	10	10	10	10
Choline bitartrate	2.5	2.5	2.5	2.5	2.5
Analyzed ^$^					
Protein (g/100g)	19.8 ± 0.3	20.0 ± 0.2	40.6 ± 0.5	39.5 ± 0.4	39.6 ± 0.4
Energy (kcal/g)	4.3 ± 0.2	5.8 ± 0.2	5.7 ± 0.3	5.7 ± 0.3	5.8 ± 0.2

^1^ Low-fat (LF), ^2^ high-fat/high-sucrose (HF/HS), and ^3^ high-fat/high-protein (HF/HP) diets. # amount added to the diets to equal 200 g crude protein/kg in the LF diet (33 E% protein) and HF/HS diet (16 E% protein) diets, and 400 g crude protein in HF/HP diets (35 E% protein). *, ** Calculated amount of crude protein * and fat ** from the protein source per kg diets. ^†^ Mineral mix: SDS—Special Diets Services AIN93G. ^‡^ Vitamin mix: SDS—Special Diets Services, AIN93VX NCR95 compliant. ^$^ Numbers represent the mean ± SEM of triplicate measurements.

## Supplementary Materials

The following are available online at https://www.mdpi.com/2072-6643/12/10/3038/s1, Figure S1: Weight of adipose tissue depots at termination.
