# Supplementary material for: The Anti-Obesogenic Effect of Lean Fish Species Is Influenced by the Fatty Acid Composition in Fish Fillets
Source: Nutrients. 2020 Oct 3;12(10):3038. doi: 10.3390/nu12103038 (PMC7600456; doi:10.3390/nu12103038)
Supplement: Supplementary file 1 [file nutrients-12-03038-s001.pdf]

Figure S1:

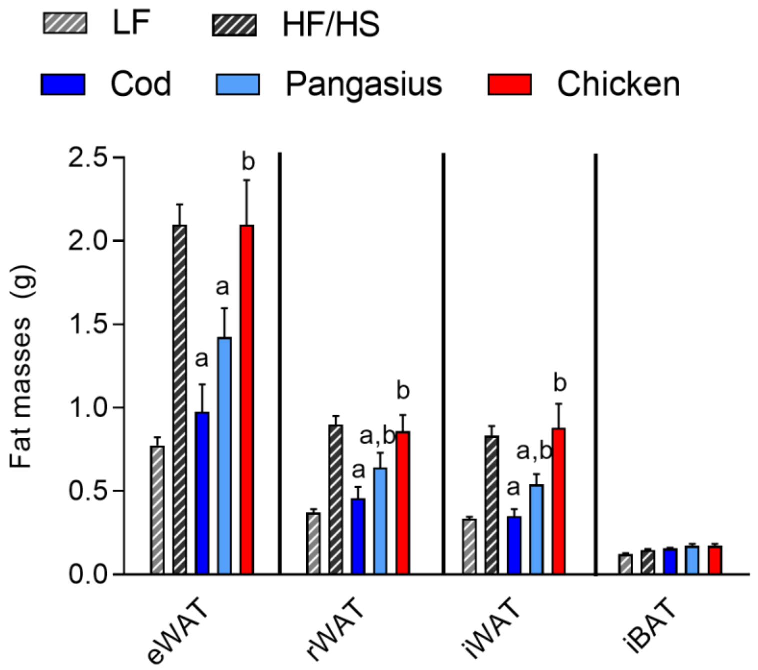

Figure 1. Weight of adipose tissue depots at termination. Data are presented as mean ± SEM (n=10) and different letters denote significant differences (P<0.05) by one-way ANOVA using uncorrected Fisher's LSD multiple comparison.
